# Evolution mechanism of water-conducting fractures in overburden under the influence of water-rich fault in underground coal mining

**DOI:** 10.1038/s41598-024-54803-5

**Published:** 2024-03-01

**Authors:** Cao Zhengzheng, Yang Xiangqian, Li Zhenhua, Du Feng

**Affiliations:** 1 International Joint Research Laboratory of Henan Province for Underground Space Development and Disaster Prevention, School of Civil Engineering, Henan Polytechnic University, Jiaozuo, 454000 Henan China; 2 Henan Mine Water Disaster Prevention and Control and Water Resources Utilization Engineering Technology Research Center, Henan Polytechnic University, Jiaozuo, 454000 Henan China; 3 Collaborative Innovation Center of Coal Work Safety and Clean High Efficiency Utilization, Jiaozuo, 454000 Henan China

**Keywords:** Underground coal mining, Fault, Overburden failure, Water-conducting fracture, Numerical simulation, Coal, Civil engineering

## Abstract

Based on the 7618 working face in Yaoqiao coal mine of Datun mining area, the activation mechanism of water-rich faults and the development characteristics of water-conducting fractures in overlying strata under the influence of faults are studied by theoretical analysis, numerical simulation and field measurement in this paper. The research results show that Anderson model and Mohr–Coulomb strength criterion are combined to establish the fault failure mechanical model, and the fault activation criterion under the influence of mining is obtained. FLAC3D numerical simulation results show that with the advance of the working face, the fault begins to be affected by the mining effect of the working face at the distance of 20 ~ 30 m from the fault. Meanwhile, with the advance of the working face, the overburden shear failure range also expands, and the fault fracture gradually expands from top to bottom. The failure zone of the working face roof is connected with the fault fracture zone. Then the fault is "activated" and causes the fault to become a water gushing channel, and finally the water gushing disaster occurs. Through numerical simulation and comparative analysis, the development height of water-conducting fracture is 73.2 m in the absence of fault, and 73.7 m in the presence of fault, indicating that the fault has little influence on the maximum development height of water-conducting fracture. The actual development height of the water-conducting fracture zone in the 7618 working face is 73.97 m and the fracture production ratio is 13.7. The research results can provide theoretical reference for the safe mining of similar working faces across faults.

## Introduction

In modern coal mine engineering, the stability of working face is the key to ensure safe and efficient mining production. Yaoqiao coal mine is located in a complex geological environment, and its unique water-rich fault has provided a significant challenge to the safety of coal mine. Especially under the influence of faults, the evolution law of water-conducting fracture in overlying strata of working face has become an urgent problem to be solved. As a part of geological structure, fault has a profound influence on the hydro-geological conditions of coal mine. In Yaoqiao coal mine, the existence of water-rich faults significantly increases the risk of water damage. In the process of coal mining, fault activities can lead to the dynamic change of water-conducting fractures in overlying strata, which affects the drainage system and the overall stability of the mine.

Domestic and foreign scholars have conducted a large number of theoretical studies on the development law of water-conducting fractures in overlying strata under the influence of water-rich faults^1–5^. Cao et al.^6^ adopted the void ratio to reflect the void or volume change of fractured rock mass in the deformation process, and established the damage model of fractured rock mass, and conducted the in-depth research on the relationship between effective stress and macroscopic nominal stress of fractured rock mass. Statistical damage theory was introduced to establish the statistical constitutive model of strain softening damage of fractured rock mass. Wang et al.^7^ used the theory of damage mechanics to comprehensively consider the coupling effect of rock mass structure and load, and established the damage evolution model and damage constitutive model of jointed rock mass. Wang et al.^8^ established a physical model of upper and lower mining in a test mine, and analyzed the dynamic evolution and mechanism of karst roof water inrush under different mining sequences. Li et al.^9^ studied the deformation activation and water conduction mechanism of faults under the influence of mining, and believed that fracture deformation activation and water conduction have obvious spatio-temporal effects. Based on the rock mass limit equilibrium theory, Zhang et al.^10^ derived the ultimate water pressure of the floor water-barrier, and established the mining stress mechanics model under the influence of faults, and obtained the mechanical criterion of fault activation under the influence of mining. Wang et al.^11,12^ showed that the cyclic application of liquid nitrogen cooling promoted the formation of internal fractures in granite and its impact on the weakening characteristics of the mechanical properties of granite. Weile et al.^13^ studied the micro-pore characteristics of remolded loess and undisturbed loess at different resting periods, indicating that the prepared loess samples have obvious thixotropy, which has certain guiding significance for improving the geotechnical properties. Xue et al.^14^ studied the fracturing effect of water-cooled impact on high-temperature rocks, indicating that water-cooled impact can significantly reduce fracture initiation pressure and induce more secondary fractures.

In terms of numerical simulation research, Li et al.^15^ studied the development characteristics of water-conducting fractures in coal mining under water body by combining deformation analysis and numerical simulation, and believed that soft rock with a certain thickness in overlying strata inhibits the upward development of water-conducting fracture zone. Xu et al.^16^ used similar simulation experiments to study the development law of upper and lower wall fractures in the working face over normal faults and reverse faults. Zeng^17^ and Wu^18^ used the nonlinear finite element numerical simulation method to study the influence of faults with different inclination angles on the height of water-conducting fractures in fully mechanized caving face, and obtained four development laws of water-conducting fractures with two inclination angles and different advancing modes. Zhao^19^, Min^20^ and Yu^21^ analyzed the influence of fault size, number and different distribution on the overburden fracture development by using similar simulation and RFPA numerical simulation methods, and found that faults located near the incision holes had a greater impact on the overburden fracture failure. Wang^22^ and Zheng^23,24^ studied the fault activation rule under the influence of mining by means of numerical simulation and theoretical analysis, and found that the shear stress drop value increased with the release of energy when fault failure occurred, the fault at the high roof was activated firstly but released less energy, and the roof and floor near the coal seam were damaged later but released more energy. Sun Wenbin et al.^25^ used similar simulation and numerical simulation to analyze the stress disturbance rule of mining stress on fault fracture zone, and used grouting to inhibit the development of fracture zone, so that the fault was not easy to activate. Cao et al.^26–28^^**.**^established a numerical model of slurry diffusion in veneer fractures, and further studied the diffusion law of grouting slurry in fractures under different rheological indexes and different consistency indexes. Liu et al.^29^^**.**^conducted a numerical study on the multi-physical field coupling mechanism in the process of microwave thermal recovery of shale gas, which provided a necessary theoretical guidance for the field application of microwave thermal recovery of shale gas.

At present, many scholars have carried out a series of studies on the fault activation mechanism and the development characteristics of water-conducting fractures in overlying strata under the influence of faults, which has an important theoretical reference significance for this study. However, the development characteristics of water-conducting fractures in overlying strata under the influence of water-rich faults through the working face in Yaoqiao coal mine area are not researched. Therefore, based on the hydro-geological conditions of Yaoqiao coal mine, mechanical model, numerical simulation and on-site monitoring are adopted to study the fault activation mechanism and the evolution law of water-conducting fractures in overburden strata under the influence of faults, which has an important theoretical significance and engineering application value to provide theoretical support for the safe mining of over-water-rich faults in similar working faces.

## Geological conditions and mining conditions

**Figure 1:**
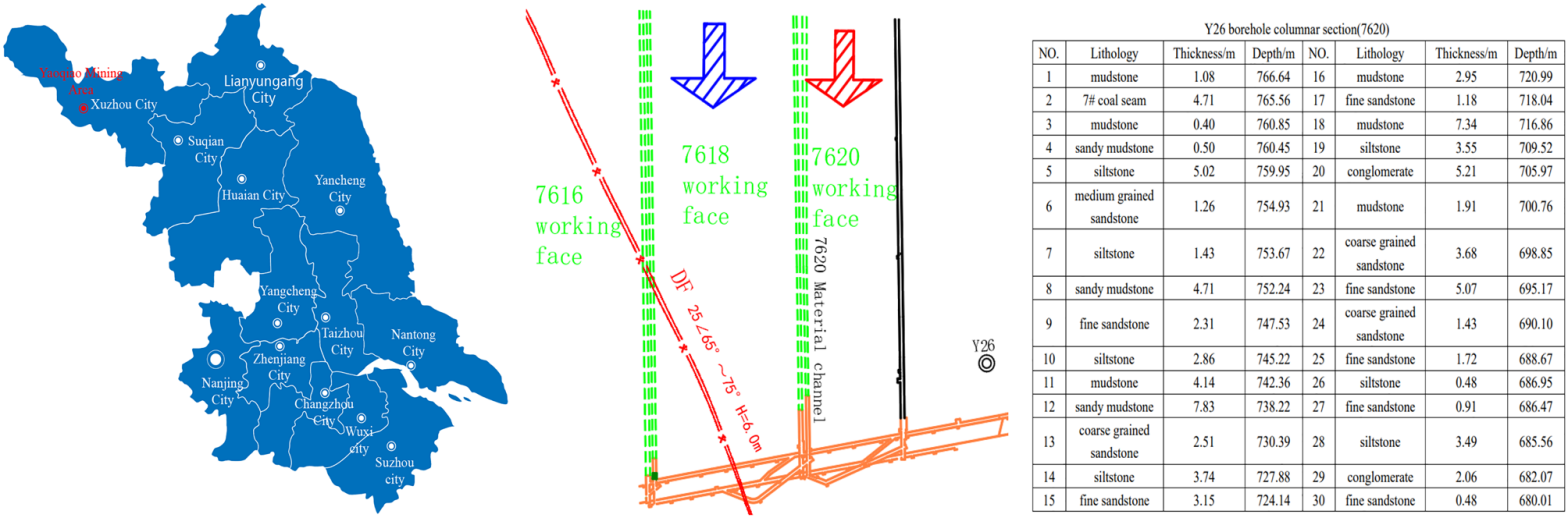
Schematic diagram of the position of the working face.

Yaoqiao coal mine is located in Xuzhou city, Jiangsu province, China. The 7618 working face in Yaoqiao coal mine is adjacent to the 7620 working face to the north and the 7616 working face to the south. The buried depth of the working face is 660 m ~ 715 m. The strike length of the working face is 1035 m, the dip length is 180 m, and the average seam thickness is 5.6 m. In the mining process of the 7618 working face, DF25 normal fault with a dip angle of 70° and a drop of 6 m is encountered, which has no influence on the safe mining of the working face. The position of the working face, the position of the fault and the column shape are shown in Fig. 1.

## Activation mechanism of water-rich fault

**Figure 2:**
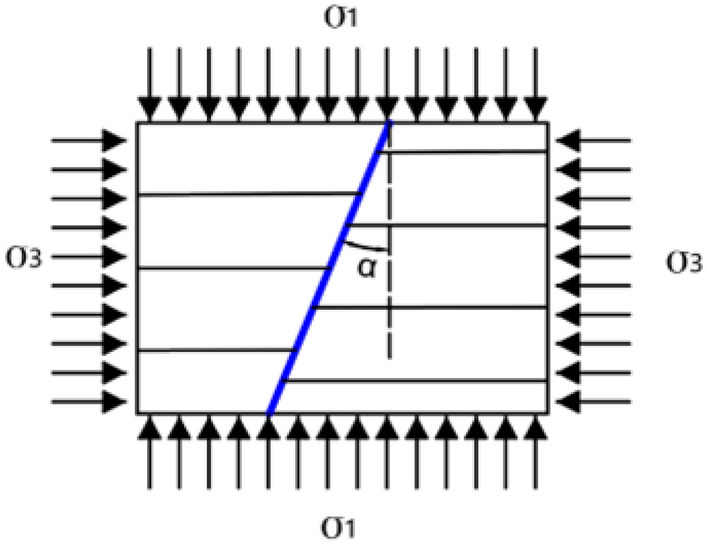
Anderson fault model.

According to the research results in literature^30,31^, it is concluded that the mining of working face has a significant impact on the stability of the fault. The change of stress state in the fault determines whether the fault is activated, and the change of stress state is the result of the redistribution of in-situ stress caused by the disturbance of mining of working face. In order to better understand the stability of faults under mining disturbance, Anderson model^32^ is adopted to effectively capture the relationship between ground stress and fault stability after the fault is disturbed by the mining of working face. The Anderson fault model is shown in Fig. 2.

σ_1_ and σ_3_ are the vertical and lateral stresses of the fault under the influence of mining effect, and α is the fault inclination, which is in the range of (0, π/2).

According to the stress state analysis of the points, the stress on the fault plane is

1 $$\sigma = \frac{{\sigma_{1} + \sigma_{3} }}{2} - \frac{{\sigma_{1} - \sigma_{3} }}{2}\cos 2\alpha - p_{i}$$

2 $$\tau = \frac{{\sigma_{1} - \sigma_{3} }}{2}\sin 2\alpha$$

where σ is the normal stress on the fault surface, MPa; τ is the shear stress on the fault surface, MPa; *p*_*i*_ is the fault pore pressure, MPa.

According to Formulas (1) and (2), it can be obtained

3 $$\left( {\sigma - \frac{{\sigma_{{^{1} }} + \sigma_{{^{3} }} - 2p_{i} }}{2}} \right)^{2} + \tau^{2} = \left( {\frac{{\sigma_{{^{1} }} - \sigma_{{^{3} }} }}{2}} \right)^{2}$$

**Figure 3:**
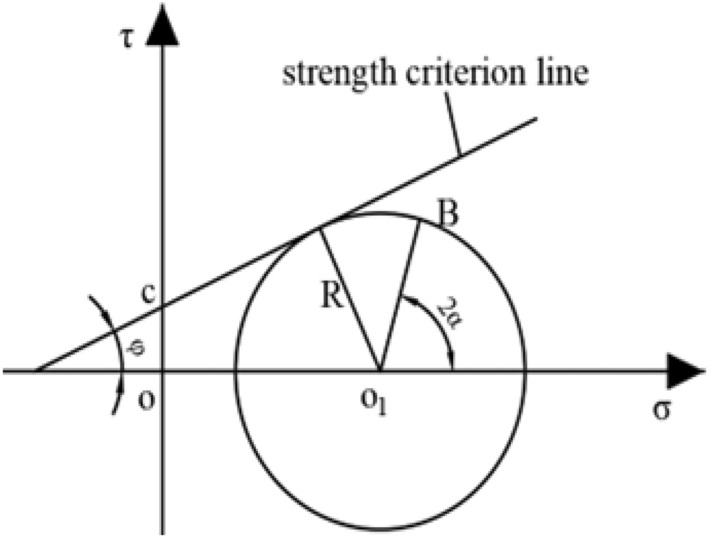
Mohr stress circle of fault plane.

The Mohr stress circle is shown in Fig. 3. The O_1_ coordinate is ((σ_1_ + σ_3_ − 2pi/2), 0), and the radius R is (σ_1_ − σ_3_) /2. The stress on the fault plane corresponds to the coordinate of point B on the Mohr stress circle.

When the fault is in equilibrium state, the normal stress σ and the shear stress τ satisfy the Mohr–Coulomb strength criterion.

4 $$\tau = c + t\sigma$$

5 $$t = \tan \varphi$$

where *c* is the cohesion of the fault plane, MPa; *t* is fault friction factor; *φ* is the internal friction angle of the fault, φ ∈ (0, π/2), rad.

Formulas (4) and (5) show that the greater the friction angle is, the greater the friction factor *k* is, the greater the shear stress *τ* is, and the higher the shear strength is.

Based on the results of previous studies^33,34^, the analysis shows that the internal friction angle changes little after fault failure, but the cohesion decreases significantly. Therefore, the slope of the strength criterion line on the fault plane remains basically unchanged; due to the influence of mining, the cohesion *c* on the fault plane decreases, which causes the whole criterion line to move downward.

**Figure 4:**
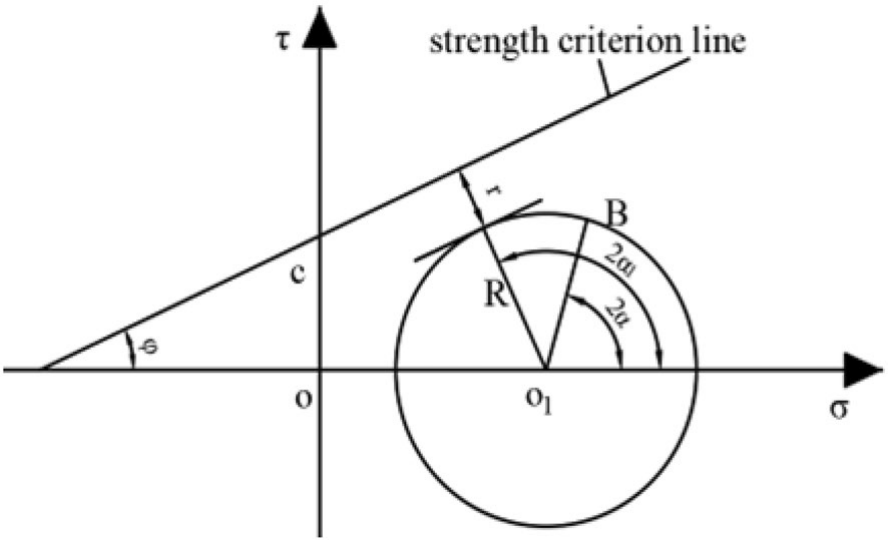
Schematic diagram of fault state identification.

Under the influence of mining, the minimum distance between the Mohr stress circle at a certain point on the fault surface and the Mohr–Coulomb strength criterion line on the fault surface is *r*, and the fault state determination diagram is shown in Fig. 4.

From the geometric relationship shown in Fig. 4,

6 $$r = (c\cot \varphi + \overline{{OO_{1} }} )\sin \varphi - R$$

Substituting the relationship between $\overline{{OO_{1} }}$ and *R*,

7 $$r = c\cot \varphi + \frac{A}{2}\sin \varphi - \frac{{\sigma_{1} - \sigma_{3} }}{2}$$

Under the influence of mining in the working face, the Mohr–Coulomb strength criterion line moves down as a whole. As shown in Fig. 4, there are three relationships between the strength criterion line and the stress circle, namely, there are three fault states.

1. When *r* > 0, the fault is in a stable equilibrium state.
2. When *r* = 0, the fault is in the limit equilibrium state.
3. When *r* < 0, the fault is in activated state.

## Evolution law of overburden fracture

### Mining fracture evolution law of non-tectonic overlying strata

#### Numerical calculation model

**Figure 5:**
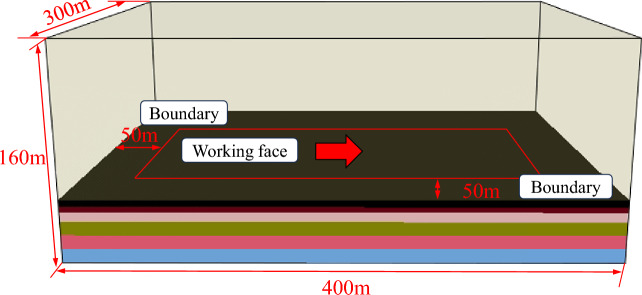
3D numerical calculation model.

Based on the geological conditions of the 7618 working face in Yaoqiao coal mine, FLAC3D numerical simulation software is used to study the development law of the water channel of the working face. The model size is 400 m × 300 m × 160 m (X × Y × Z). In order to eliminate the boundary effect, 50 m boundary coal pillars are established before and after the working face strike direction, and 50 m boundary coal pillars are established on both sides of the inclined direction. The advancing direction of the working face is set to 300 m, and the numerical model is shown in Fig. 5.

The vertical initial stress of the simulation model is calculated according to the weight of the overlying strata, and the average volume force of the overlying strata is 24 kN/m^3^. According to the buried depth of 700 m, the vertical initial stress is set at 16.7 MPa. Therefore, the vertical load of 16.7 MPa is applied to the upper part of the model to simulate the weight load of the overlying strata, and the horizontal initial stress is set at 20.87 MPa. There are fixed constraints around and on the bottom of the model. The construction of the numerical model is based on drill hole *y*-26. The mechanical parameters of coal and rock mass refer to the relevant values in Yaoqiao coal mine, and the mechanical parameters are shown in Table 1.

**Table 1:** Calculation parameters of the numerical model.

Lithology	Thickness/m	*γ*/(kN/m^3^)	*E*/GPa	u	*T*/MPa	*c*/MPa	*φ*/°
Coarse grained sandstone	2.51	2280	5.65	0.26	30.22	4.6	35
Fine sandstone	2.31	2580	7.62	0.36	83.63	7.53	30.11
Medium grained sandstone	1.26	2716	6.64	0.32	37.77	7.47	34.11
Siltstone	5.02	2540	5.10	0.32	44.71	6.32	33.65
Sandy mudstone	0.5	2590	7.57	0.24	64.56	3.8	34.12
Mudstone	0.4	2372	3.14	0.32	19.54	4.39	30.59
7# coal seam	4.71	1283	2.5	0.28	7.16	6.32	24.29
Mudstone	1.08	2372	3.14	0.32	19.54	4.39	30.59

#### Failure, movement and evolution law of overlying strata

##### Evolution law of leading bearing stress

**Figure 6:**
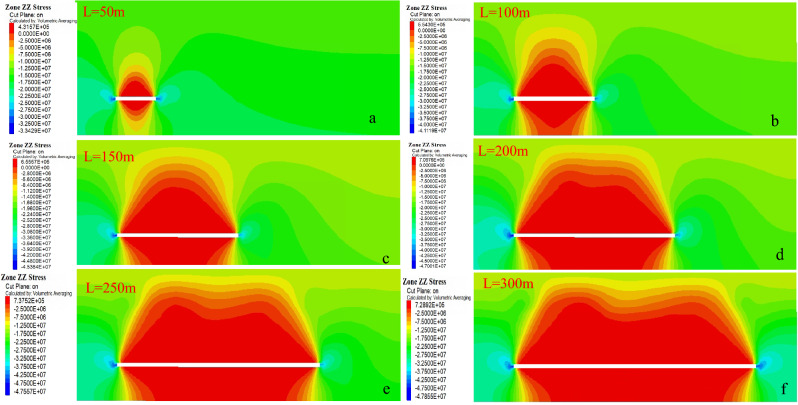
Distribution of leading bearing stress on the working face.

The overlying strata is subjected to continuously changing disturbance stress due to coal mining. When the rock stress exceeds its elastic–plastic bearing force, it breaks and produces water-conducting fractures. Therefore, based on the analysis of the stress change law of overlying strata, the current state of overlying strata can be indirectly reflected, and then how the stability of overlying strata is affected by mining is analyzed. The change of stress state in stope is shown in Fig. 6.

When the working face advances to 50 m, the surrounding rock stress along the coal seam makes the pressure relief arch with a small range appear in the upper part of the goaf. The vertical stress is symmetrically distributed along the central axis of the goaf, and the bottom floor of the goaf also forms a pressure relief zone. It shows that there are caving or separation fractures in the overburden in this area, and the stress concentration is generated near the coal wall and near the palm face in front of the working face, the maximum is 34 MPa. When the working face advances to 100 m, the mining disturbance presents a more significant influence, and the relief arch gradually extends upward; the stress concentration is significant, and the stress value increases. When the working face advances to 200 m and 250 m, the overall change trend of stress is basically similar to the position of the working face to 150 m. Through continuous advancement of the working face, the pressure relief arch continues to expand, and the concentrated stress is still near the coal wall of the opening hole and near the palm face in front of the working face. When the working face advances to 300 m, the contact between the top and bottom areas causes the stress value in the middle of the goaf to change; the stress concentration in the area before and after the goaf is relieved, and the stress concentration value decreases, and the relief arch gradually self-differentiated at this time. With the collapse, subsidence and compaction of the overlying strata, the pressure in the middle relief area slowly recovers, and the stress in the overlying strata in the goaf slowly becomes stable.

##### Variation rule of overlying strata displacement

**Figure 7:**
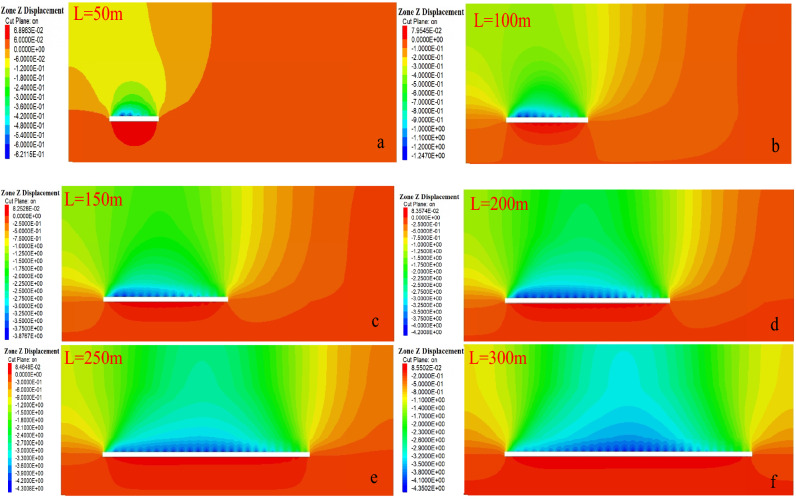
Change of vertical displacement of working face.

In order to study the displacement deformation of overlying strata after coal mining in the working face, the overall evolution process of water-conducting fracture zone is indirectly reflected by analyzing the change of vertical displacement of overlying strata in the stope. The change of vertical displacement of the working face is shown in Fig. 7.

As shown in Fig. 7, subsidence displacement occurs in overlying strata above the goaf at 50 m, and the displacement cloud map forms a small "arch" shape. Since it is the initial mining stage, subsidence of overlying strata is not large, and the maximum subsidence of direct roof and basic roof is 0.52 m. Floor heave occurs in goaf floor due to the compression of bearing overlying strata on both sides, and the floor heave value is about 0.101 m. When the working face advances to 100 m, the uneven arch deformation zone appears in the middle rock layer above the coal seam, which is caused by the uneven settlement of the roof and roughly deviates to the position of the cutting hole. The displacement of the basic roof and the direct roof is large. When the working face advances to 150 m, 200 m and 250 m, the overall movement and deformation trend of the overlying strata is basically the same as that at 100 m when the working face advances to 100 m, and the "arch" of the displacement cloud map of the overlying strata in the goaf continues to increase. When the working face advances to 300 m, the subsidence value of overlying strata in the goaf continues to increase, and the sum of the subsidence of the basic roof and the floor heave value of the coal seam is close to the mining height; the mining state is basically sufficient, and the development of water-conducting fractures is basically stable at this time. It is obvious that the overall movement and deformation range of overlying strata continue to expand due to continuous advancement of the working face. If the working face advances to a corresponding distance, the overall increase of vertical movement and deformation of overlying strata continue to decrease and become increasingly stable, indicating that the development height of water-conducting fractures has become stable.

##### Variation rule of plastic zone in overlying strata

**Figure 8:**
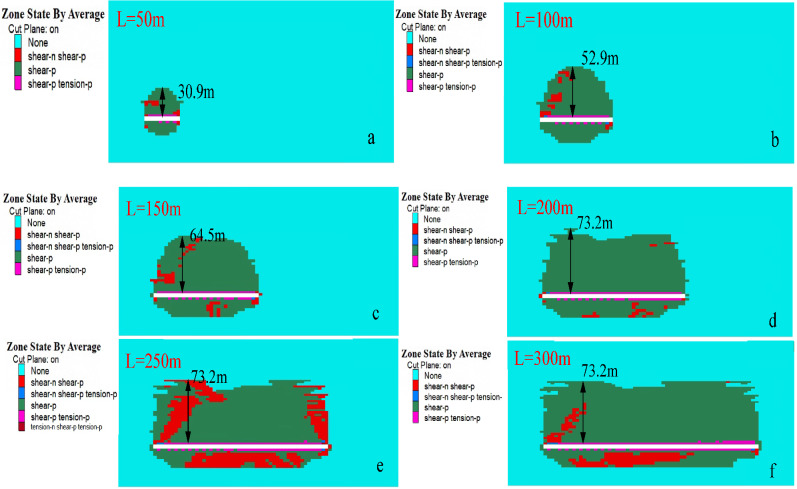
Variation of plastic zone of excavation in strike direction.

Selecting the distribution characteristics of plastic zones at different distances of excavation in the direction of the model, shown in Fig. 8, and observing the damage zone caused by excavation of the working face to the roof strata.

**Figure 9:**
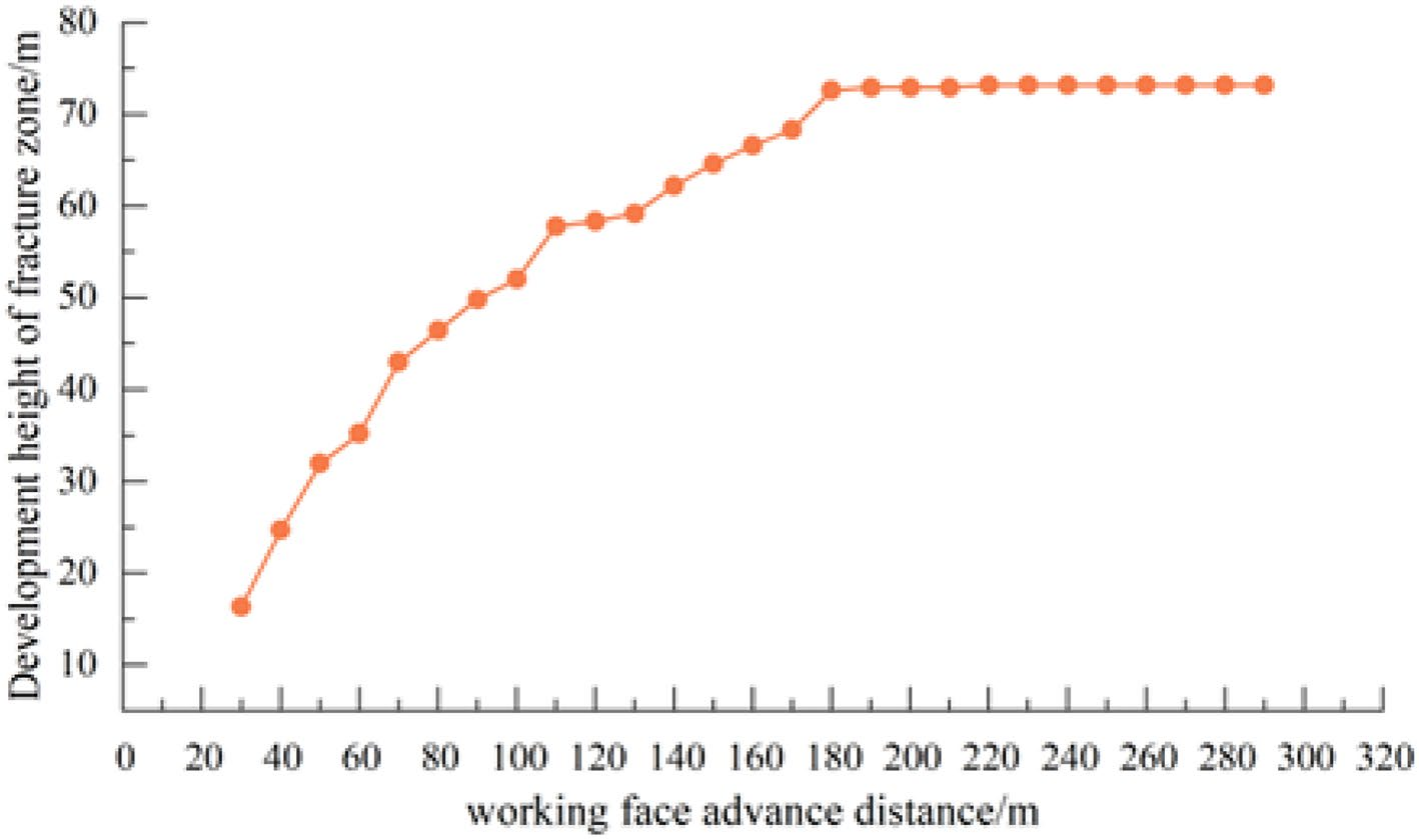
Development height of water-conducting fracture zone.

After coal seam excavation, the plastic zone of overlying strata develops to the depth. After excavation of coal seam is 50 m, the maximum failure height of the plastic zone is 30.9 m; and when the working face advances to 100 m, the development range of the plastic zone expands with the advancement of the working face. Shear failure and tensile failure in the direct roof occur simultaneously, and shear failure of deep overlying strata continues. This indicates that the maximum development height of the water-conducting fracture at this time is 52.9 m. With the continuous expansion of mining scope, the influence range of mining expands. By comparing the distribution of the model plastic zone in the strike of 200 m, 250 m and 300 m, when the working face is excavated to 200 m, the scope of the model plastic zone continues to expand along the strike direction, but the plastic zone in the model Z direction no longer develops upward. At this time, the overlying strata on the working face has reached full mining movement. The fracture zone of water conduction reaches its maximum height. The distance of coal seam roof coordinate and the maximum height coordinate of the plastic zone are calculated as the development height of the water-conducting fracture zone, and the calculation results are shown in Fig. 9.

According to the calculation results, the deformation and failure of the overlying strata in the goaf develop gradually from bottom to top. Firstly, caving zone is formed in the lower rock layer, and then fractures and expansion occur in the middle rock layer. With the continuous expansion of mining scope, the fractures in the overlying strata further develops. After the working face advances to a certain distance, the height of water-conducting fracture zone gradually becomes stable, and the final development height of water-conducting fracture zone in working face is 73.2 m.

### Analysis of mining fracture evolution law of fault-bearing overlying strata

#### Numerical calculation model

**Figure 10:**
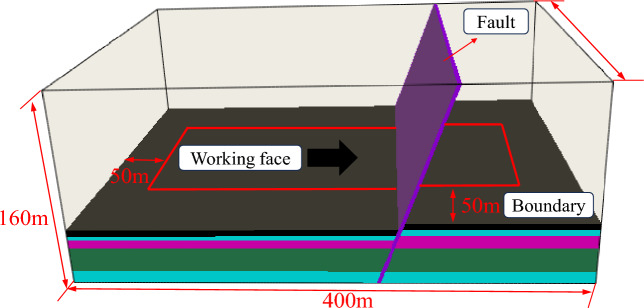
3D numerical calculation model.

The size of the numerical model established is 400 m × 300 m × 160 m (X × Y × Z). In order to eliminate the boundary effect, 50 m boundary coal pillars are established before and after the strike direction of the working face, and 50 m boundary coal pillars are established on both sides of the trend direction, and 300 m is set as the advancing direction of the working face. The numerical model is shown in Fig. 10.

Starting from 200 m away from the fault, the working face is divided into six stages according to engineering experience, which are 70 m away from the fault, 30 m, 20 m, 10 m, 0 m and – 10 m. Based on the analysis of the advance bearing stress of calculation results, the displacement and failure state of plastic zone of the working face at different distances from the fault, and the law of mining engineering effect on mining fracture of overburden rock under the influence of fault geological structure are analyzed.

#### Variation law of leading bearing stress of working face

##### Evolution law of leading bearing stress of working face

**Figure 11:**
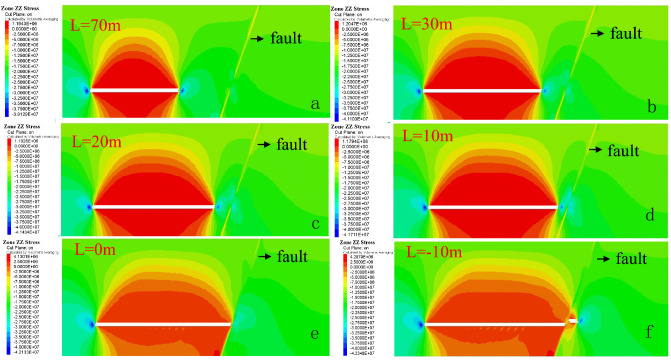
Distribution of leading bearing stress on the working face.

**Figure 12:**
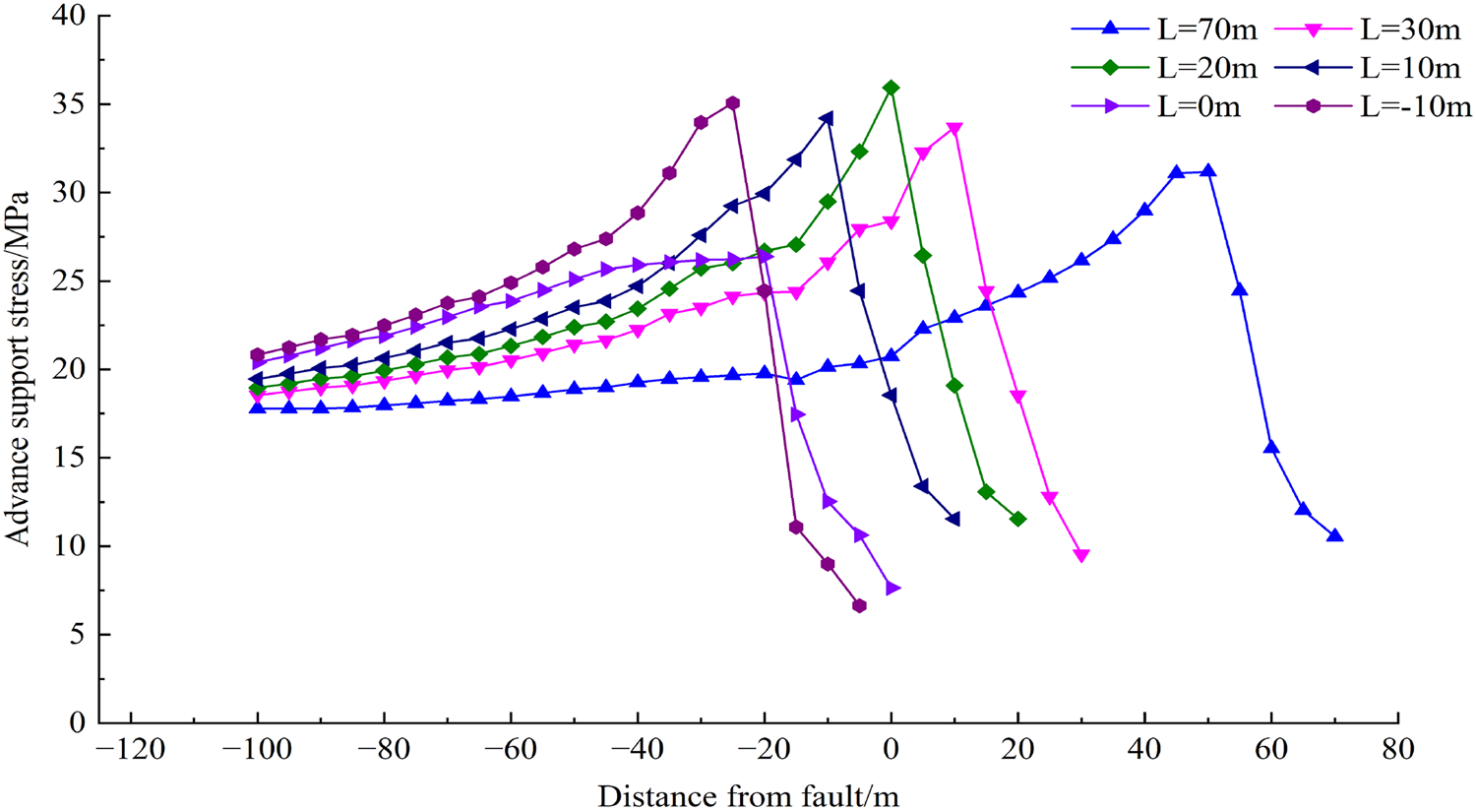
Curves of leading bearing stress at different stages of working face.

Through numerical simulation calculation, the distribution characteristics of the leading supporting stress at different distances from the fault under the influence of the fault are simulated and analyzed. The distribution diagram of the leading supporting stress at the working face is shown in Fig. 11, and the change curve of the leading supporting stress is shown in Fig. 12.

With the advancement of the working face, the influence range of the leading supporting stress on the working face does not change significantly, and the peak value of the leading supporting stress is about 20 m in front of the working face, indicating that the influence range of the fault is about 20 m in front of the working face. When the working face advances to 30 m away from the fault, due to the continuous advancement of the working face, the leading supporting stress of the working face gradually enters the area affected by the fault, and the leading supporting stress of the working face is affected by the fault barrier effect, leading to the result that the leading supporting stress of the working face cannot continue to conduct forward and disperse. On the other hand, as the working face begins to advance along the strike towards the fault, the supporting stress in front of the working face increases and reaches the maximum when the mining length is 20 m away from the fault. The maximum leading supporting stress can reach 39.6 MPa, which is 2.3 times of the initial stress. Combined with Fig. 11, when the working face advances to 20 m, the advancing abutment pressure has already affected the fault area. When the working face continues to advance from 10 m away from the fault, the leading bearing force drops sharply to 26.4 MPa, and the stress concentration coefficient is 1.6. When the working face passes through the fault, the leading bearing stress increases again. In fact, the reason for the stress change in the above process is that the cutting action of faults divides the strata into upper and lower plates, and the advancement of working face forms a structure similar to "coal pillar" between the fault and working face. Under the combined action of mining effect and fault barrier effect, the width of coal pillar bearing the advance supporting stress in front of the working face becomes smaller and smaller. The stress becomes more concentrated and the stress peak becomes larger. As the working face continues to advance, when the coal pillar is small enough, its overall strength can not withstand the advance abutment pressure of the working face, resulting in the plastic failure of the "coal pillar"; and its bearing capacity for the overlying strata is also reduced, thus triggering the "activation" of the fault, resulting in the aquifer pouring into the working face through the fault.

##### Variation rule of overlying strata displacement

**Figure 13:**
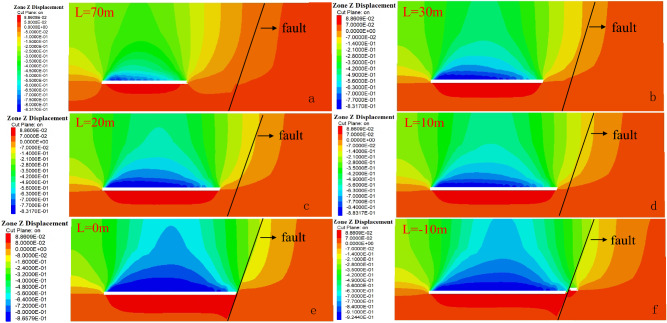
Vertical displacement distribution of overlying strata on working face.

**Figure 14:**
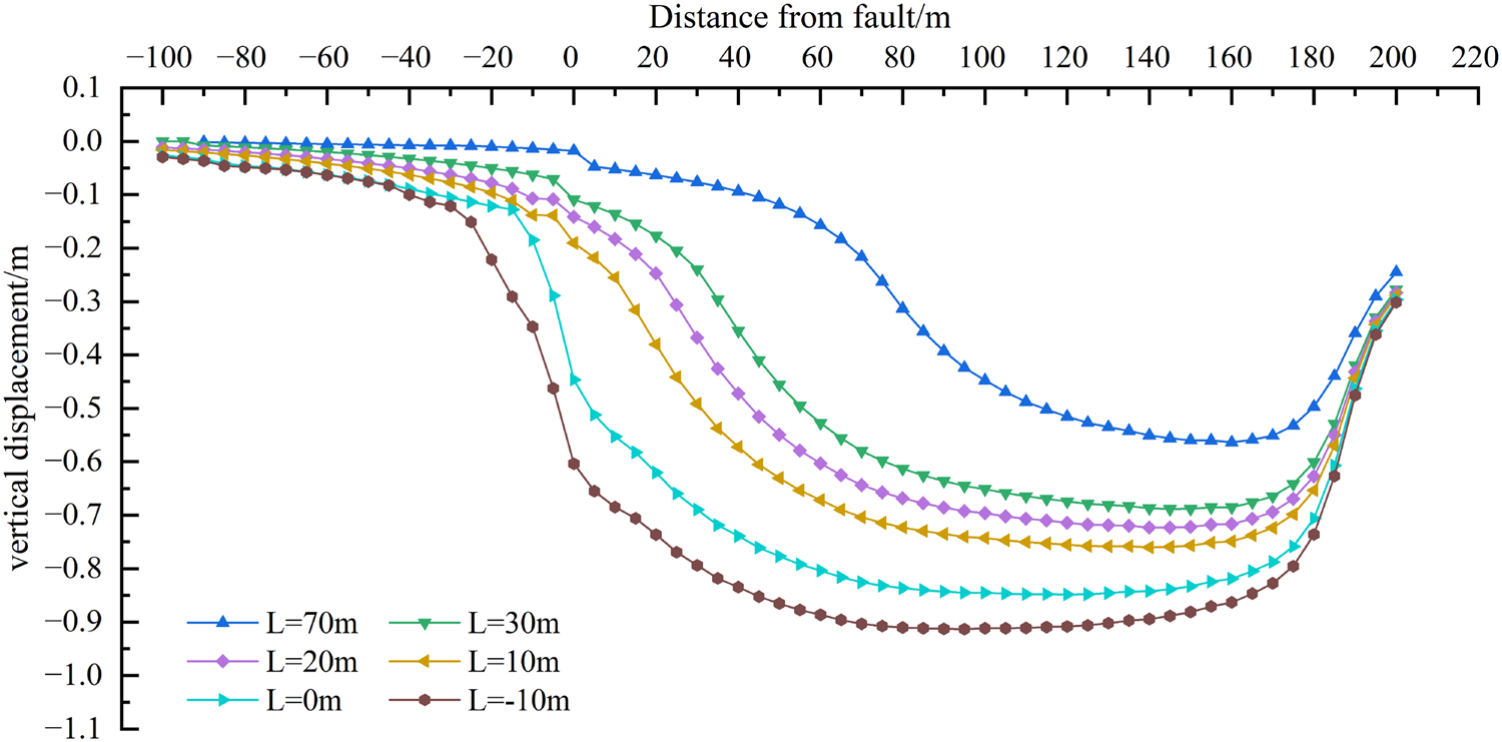
Displacement change curves of working face at different stages.

The fault geological structure has an influence on the stability and displacement of overlying strata on the working surface. In the mining activities of the working face, the stress balance of the overlying strata is damaged. Due to the action of the overlying strata load, the overlying strata further lose stability and break. In order to achieve the balance again, the overlying strata break and sink under the action of gravity load until it contacts the goaf floor and reaches the balance again. Therefore, the whole evolution process of water-conducting fracture zone can be indirectly reflected through the change of overlying strata displacement. The displacement change law of overlying strata on the stoping fault in the working face is shown in Fig. 13, and the displacement change curve is shown in Fig. 14.

When the working face begins to be mined, overlying strata above the coal seam begin to bend and sink. The area where overlying strata bend and sink gradually expands from the direct roof of the working face to the basic roof of the coal seam above the working face; the deformation scale of overlying strata becomes larger, namely, the displacement generated by overlying strata becomes larger. When the working face advances to 20 m, the affected area of displacement and subsidence gradually spreads to the fault. As the working face continues to advance, the displacement subsidence of overlying strata above the working face continues to increase, and the displacement subsidence decreases obviously when approaching and passing through the fault, and the increasing area of displacement subsidence rises firstly and then decreases.

Combined with Fig. 13, when the working face advances close to the fault or passes through the fault, the displacement and subsidence of the upper and lower plates of the fault affect the regional discontinuity, and the fault boundary is obvious. It is inferred that the roof and overburden above the working face have caved totally, forcing stress transfer, and the concentration of supporting stress in the clamping area between the fault. Therefore, the "activation" possibility of fault is also the greatest when the working face is close to the fault and passing through the fault.

##### Variation rule of plastic zone of overlying strata

**Figure 15:**
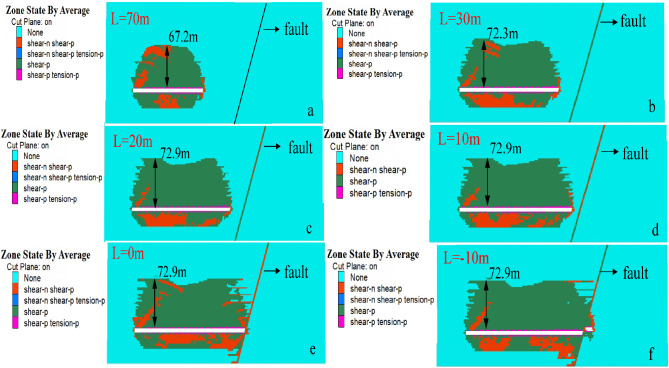
Distribution of stoping plastic zone on the working face.

**Figure 16:**
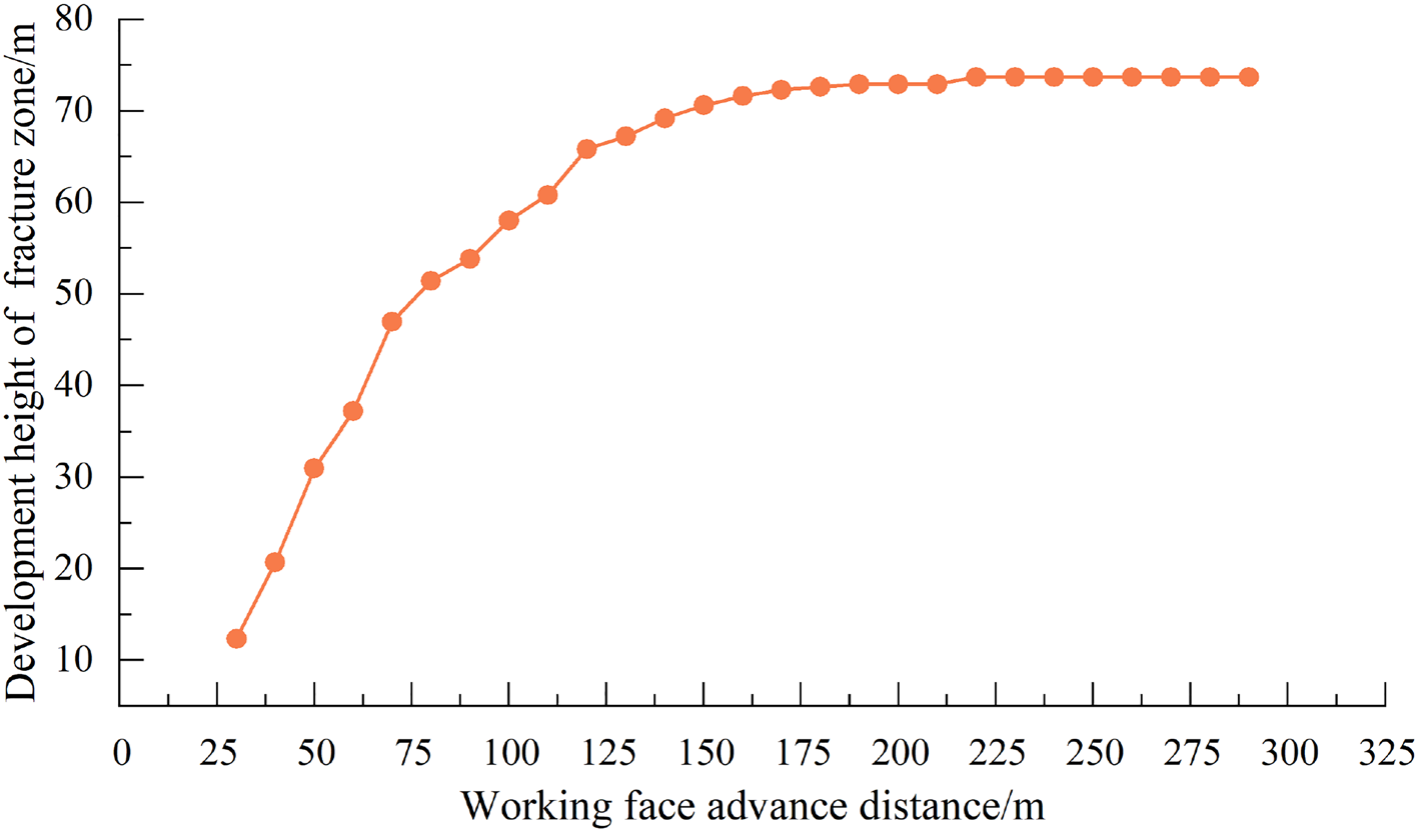
Development height of water-conducting fracture zone.

After the mining starts, the overlying strata enters a state of plastic failure under the influence of mining effect, and the existence of fault geological structure forms the condition of unbalanced stress distribution, which have a great influence on the stability of surrounding rock. At the same time, fault activation further affects the development height of the plastic failure zone, so it is necessary to analyze the plastic failure law of overlying strata in the working face to reflect the overall evolution process of the water-conducting fracture zone. The evolution law of the plastic zone during the mining of the working face is shown in Fig. 15, and the development height of water-conducting fractures in the overlying strata is shown in Fig. 16.

It can be seen from the Fig. 15 that the tension failure of the direct roof above the coal seam enters a plastic state in the initial stage of working face. The front and bottom of the working face are subjected to shear failure and enter a plastic state. The failure development height of overlying strata in the working face is shown in Fig. 16. When the working face advances to 170 m, the plastic zone of the working face presents a "saddle type" distribution, and the failure height of the overlying strata is 72.9 m, and the failure height of the overlying strata is 73.7 m at the end of the working face. When the working face is far away from the fault, the fault is not disturbed by the mining of the working face. When the mining distance of the working face is 30 m from the fault, the fault begins to be affected by mining. With the advancement of the working face, the scale of shear failure of the fault gradually increases, extending from the upper part of the fault to the lower part of the fault. When the working face advances to the fault position and passes through the fault, a large area of plastic zone appears in the rock mass of the fault foot-wall; the roof plastic failure zone of the working face penetrates through the fault fracture zone, and the plastic zone of the roof near the fault extends significantly to the fault wall; the fault becomes an "activated" slip, resulting in the fault becoming a water gushing channel.

## Measurement height of "two belts" in overlying strata

### Drilling parameters

**Figure 17:**
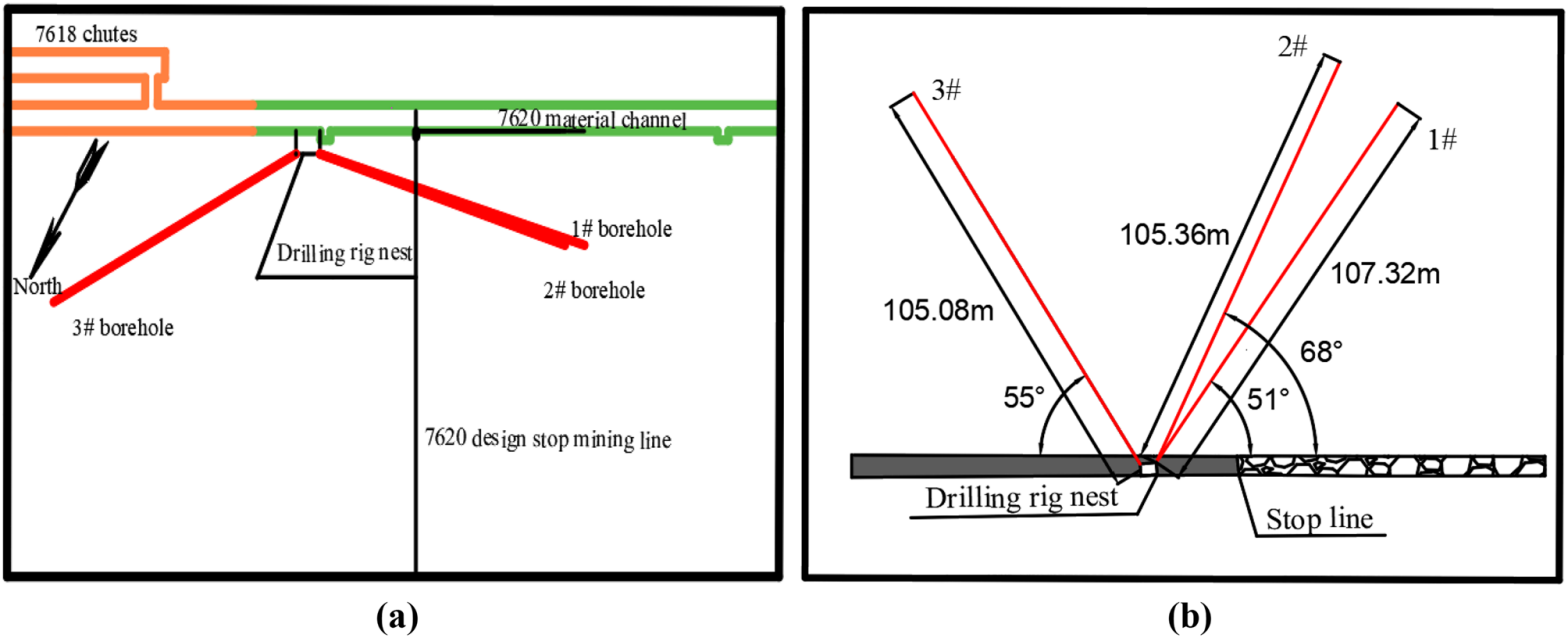
Schematic diagram of layout and profile of probe holes. (**a**) Drilling plan, (**b**) Borehole profile.

The method has the advantages of small engineering amount, low cost, high precision, and simple operation. A total of 3 boreholes are designed for exploration, among which 1 borehole is the original fracture borehole before mining (comparison borehole) and 2 borehole are the fracture development boreholes after mining. The construction drilling hole (drilling field) is arranged 5 ~ 8 m in front of C3 wire point of material track 7620, namely, 21 m outside the stop-mining line. 1# and 2# detection holes are arranged in the stoping section of the working face; 3# detection hole is arranged in the coal pillar section of the working face as a comparison hole; by comparing the measurement data before and after mining, the development height of water-conducting fracture zone in overlying strata can be accurately determined. Drilling construction parameters are shown in Table 2. The horizontal and sectional views of the probe borehole are shown in Fig. 17.

**Table 2:** Drilling construction parameters.

Rrill hole number	Dip angle/°	Azimuth/°	Hole depth/m	Vertical depth/m
1#	51	249	107.32	82.39
2#	68	265	105.36	96.93
3#	55	31	105.08	65.8

### Analysis of detection results

**Figure 18:**
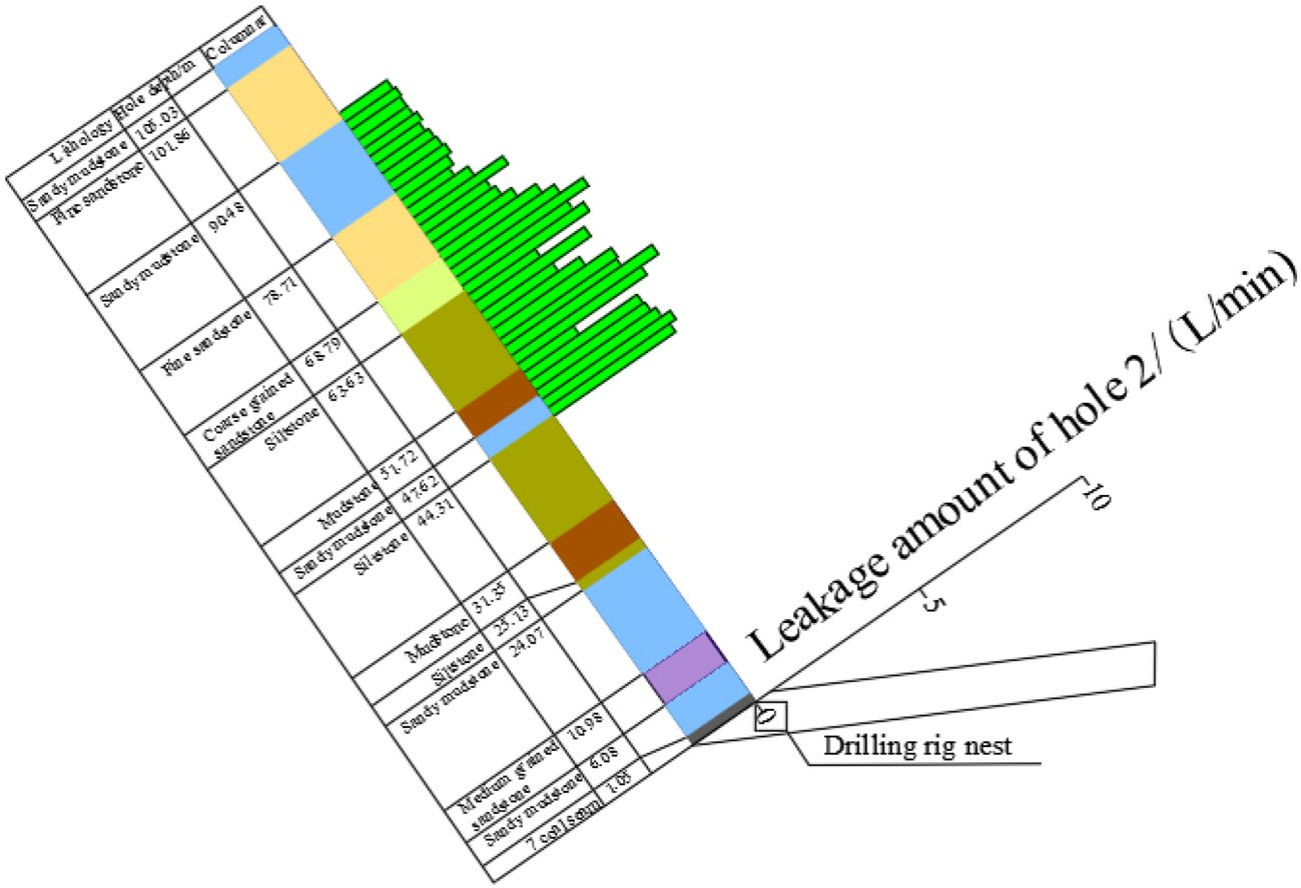
Leakage in hole 3#.

As can be seen from Fig. 18, under the condition that the overlying strata of the working face is not damaged, the average variation of water injection flow in the 3# pre-mining comparison hole is about 2.51 L/min. The rock layer around the drilling hole is not damaged by mining. According to the field observation, there are different leakage amounts in the test section of the comparison hole, especially in the 50 ~ 77 m hole section and 78.5 ~ 105 m hole section, and the water injection leakage amounts vary in the range of 1.76 ~ 4.75 L/min and 1.23 ~ 1.89 L/min, respectively.

**Figure 19:**
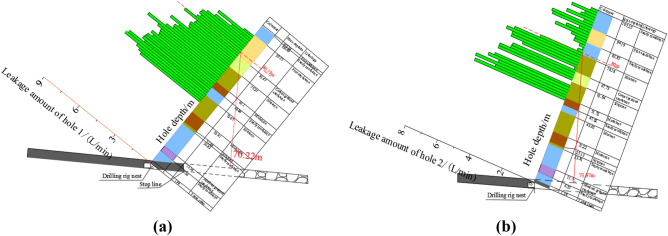
Leakage from holes 1# and 2#. (**a**) The amount of leakage from hole 1#, (**b**) The amount of leakage from hole 2#.

As can be seen from Fig. 19a, the leakage of water injection in the range of 90.5 ~ 102 m of borehole 1# fluctuates in the range of 0.8 ~ 3.75 L/min. The comparison of water leakage with the corresponding section of comparison hole No. 3 shows that the rock formation in this section is not damaged. In the range of 50 m ~ 89 m hole depth, the leakage of water injection increases, which is significantly higher than that in the previous section, and the leakage reaches 3.8–6.55 L/min, indicating that this section is the top of the water-conducting fracture zone. Therefore, the location of the overlying strata water-conducting fracture zone determined by borehole 1# is 89 m deep, the corresponding rock strata is fine sandstone of 10.45 m thickness, and the vertical height of the coal seam roof is 70.22 m.

As shown in Fig. 19b, water leakage from hole 2# fluctuates between 0.35 and 2.0 L/min at hole depths ranging from 80 to 102.5 m. The comparison with the corresponding section of hole 3# shows that the strata in this section are not damaged. The water leakage in the range of 50 m ~ 78.5 m hole depth is significantly higher than that in the previous section, and the leakage reaches 0.15 ~ 5.65 L/min, indicating that this section is the top of the water-conducting fracture zone. The rapid increase of water leakage in this section indicates that the rock formation damage is more serious from this section and enters the range of water-conducting fracture zone. Therefore, the top boundary of the water-conducting fracture zone in the overlying strata in the working face determined by hole 2# is 80 m deep, and the vertical height of the seam roof is 73.97 m.

After the end of mining, the overlying strata is affected by mining, and a large number of new fractures occur, and the leakage of borehole 1# reaches 3.8 ~ 6.55 L/min. The loss of borehole 2# is 0.15 ~ 5.65 L/min. The loss in hole 2# is generally less than that in hole 1#. This is due to the poor plugging effect of borehole 2# and the gradual closure of overlying strata fractures under the action of compaction of upper rock and loose layer after mining in the working face, so the water leakage of 2# borehole is significantly less than that of 1# borehole.

The maximum development height of water-conducting fracture zone in borehole 1# is 70.22 m, and the fracture production ratio is 13. The maximum height of water-conducting fracture zone in borehole 2# is 73.97 m, and the fracture production ratio is 13.70. Therefore, the maximum measured development height of water-conducting fracture in 7620 working face is 73.97 m, so the fracture production ratio is 13.70.

### Drilling and peering

**Figure 20:**
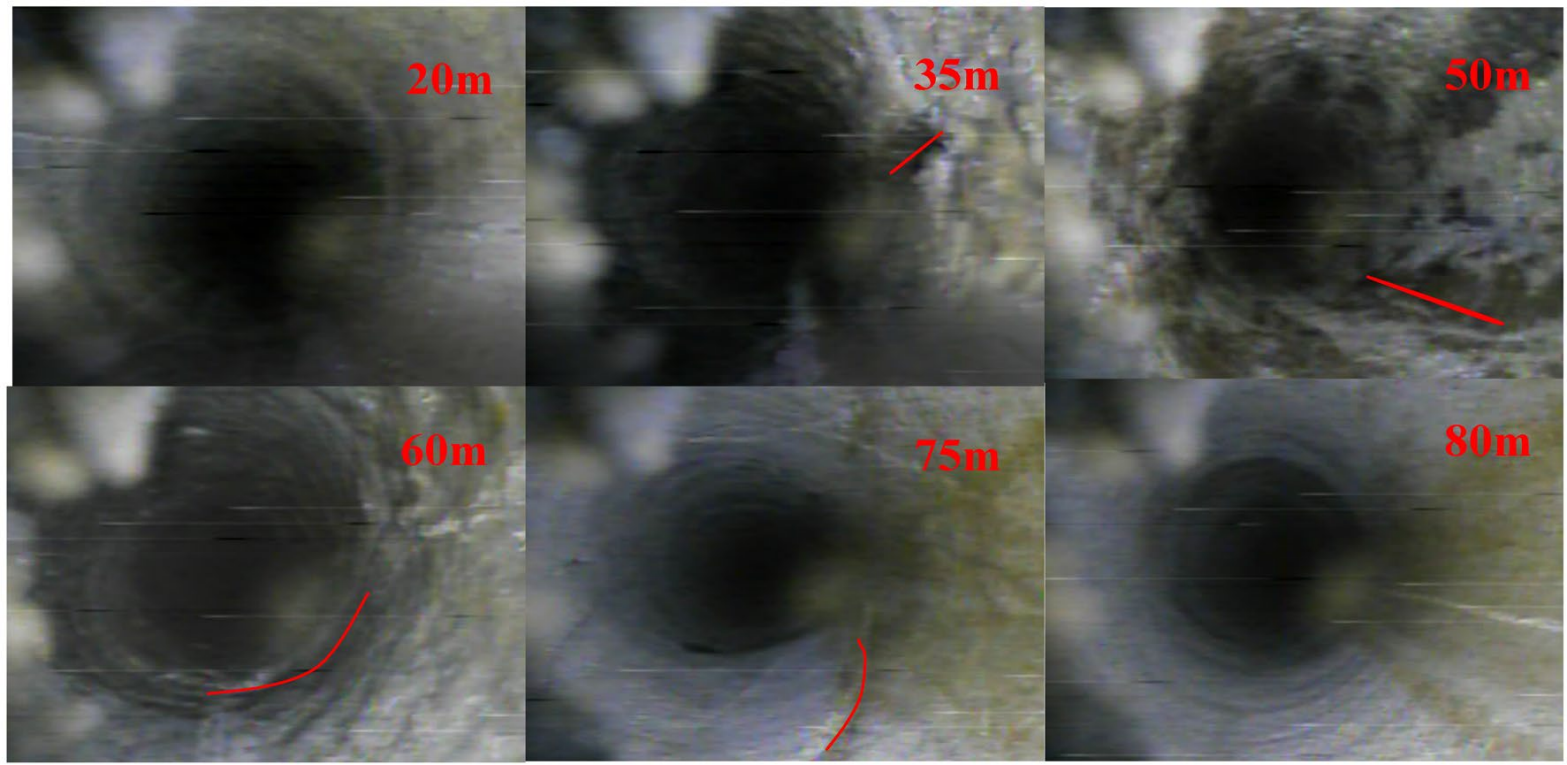
Photos of post-mining fracture.

Borehole 2# is selected as the observation hole for observation. The depth of borehole observed in the field is 105.25 m, the first 10 m includes borehole casing, and the actual effective observation depth is 95.25 m. The development of overburden fracture after mining is shown in Fig. 20.

There is no obvious mining-induced fracture in the overlying strata within 20 m before the hole depth, indicating that the hole segment in this range has not entered the fracture zone. From the hole depth of 35 m, obvious mining-induced fractures begin to appear in overlying strata, but the width and number of fractures are small, and the overlying strata are not obviously affected by mining. When the hole depth continues to increase, the discern-ability of fracture strike and width increases, and the oblique fracture is the main one, showing obvious regularity. With the increase of the advancing distance of the working face, the fracture of overlying strata decreases. When the hole depth is 80 m, namely, the vertical depth is 73.97 m, the fracture completely disappears, indicating that the height of the water-conducting fracture zone is 73.97 m. The fracture production ratio is 13.70, which is consistent with the fracture zone determined by the borehole leakage observation method. There is little change of mine water inflow during the mining process in the working face, which indicates that the compound rock fracture does not spread to the strong rich aquifer in the loose layer, and the overlying strata is "three zones" after the advancement of working face.

## Conclusions

Aiming at the development characteristics of water-conducting fracture zone in overlying strata under the influence of water-rich fault in working face, a fault failure mechanical model combined with Mohr–Coulomb strength criterion is proposed based on Anderson model. Numerical simulation and field measurement methods are used. In this paper, the overlying strata movement and the development and distribution characteristics of fractures in the working face are studied, and the evolution law of water-conducting fractures in the working face under the influence of water-rich faults is systematically clarified. The following conclusions are obtained as follows:

1. The Anderson model is employed to explain the relationship between ground stress and fault stability after the fault is affected by mining in the working face. According to the Mohr–Coulomb strength criterion analysis, the greater the internal friction angle of the fault is, the greater the friction factor is, the greater the shear stress is, and the higher the shear strength is. When *r* > 0, the fault is in a stable equilibrium state. When *r* = 0, the fault is in the limit equilibrium state. When *r* < 0, the fault is in activated state.
2. The maximum development height of the water-conducting fracture zone is 73.2 m in the absence of faults, and 73.7 m in the presence of faults, indicating that faults have little influence on the maximum development height of the water-conducting fracture zone. The influence range of the fault is 20 ~ 30 m ahead of the working face. When the working face is close to the fault, the displacement and subsidence influence area of the upper and lower plates of the fault show discontinuity. The overlying strata failure area of the working face is connected with the fault fracture zone, and the fault "activation" slip makes the fault become a water gushing channel.
3. The maximum height of the water-conducting fracture zone is 73.97 m, which is consistent with the research on the evolution law of overburden fractures, according to the results of in-situ drilling leakage detection and drilling observation. The water inrush phenomenon can be prevented when the working face passes through the fault by leaving water-proof coal pillar at the fault. In order to prevent the fault from diverting water, it is necessary to set up water-proof coal pillar or carry out advance exploration and release water. The method of grouting can be used to strengthen the weak area and reduce the probability of water inrush.

## Data Availability

Some or all data, models, or codes generated or used during the study are available from the corresponding author by request.
